# A Collection of Molecular Fingerprints of Single Aerosol Particles in Air for Potential Identification and Detection Using Optical Trapping-Raman Spectroscopy

**DOI:** 10.3390/molecules27185966

**Published:** 2022-09-14

**Authors:** Haifa Alali, Yukai Ai, Yong-Le Pan, Gorden Videen, Chuji Wang

**Affiliations:** 1 Department of Physics and Astronomy, Mississippi State University, Starkville, MS 39759, USA; 2 DEVCOM Army Research Laboratory, 2800 Powder Mill Road, Adelphi, MD 20783, USA

**Keywords:** molecular fingerprints, optical-trapping Raman spectroscopy (OT-RS), aerosol, characterization, identification, detection, single particle, native atmospheric state

## Abstract

Characterization, identification, and detection of aerosol particles in their native atmospheric states remain a challenge. Recently, optical trapping-Raman spectroscopy (OT-RS) has been developed and demonstrated for characterization of single, airborne particles. Such particles in different chemical groups have been characterized by OT-RS in recent years and many more are being studied. In this work, we collected single-particle Raman spectra measured using the OT-RS technique and began construction of a library of OT-RS fingerprints that may be used as a reference for potential detection and identification of aerosol particles in the atmosphere. We collected OT-RS fingerprints of aerosol particles from eight different categories including carbons, bioaerosols (pollens, fungi, vitamins, spores), dusts, biological warfare agent surrogates, etc. Among the eight categories, spectral fingerprints of six groups of aerosol particles have been published previously and two other groups are new. We also discussed challenges, limitations, and advantages of using single-particle optical trapping-Raman spectroscopy for aerosol-particle characterization, identification, and detection.

## 1. Introduction

Aerosol particles are microscopic solids or liquids that are from mineral dust, carbon black, metal flakes, soot, sea spay, or biological organisms including fragments and microorganisms, with a size ranging from 0.01 µm to 100 µm [1,2]. Aerosol impacts human health, climate, and biological security. For example, the aerosol pollution that derives from industrial emissions, biomass burning, dusts, etc., causes huge impacts on human health, weather, tropospheric oxidation, etc. [3]. Some aerosolized fungi or bacteria in the atmosphere, such as Aspergillus, Penicillium, and Streptococcus, have been associated with various respiratory allergies or lung diseases, such as asthma, rhinitis, and hypersensitivity pneumonitis [4,5]. In addition, the aerosolized bacteria and fungi, due to their biological metabolites and interactions, can aggressively affect cloud formation and ice nucleation in the atmosphere [6,7]. Furthermore, some life-threatening substances such as biological warfare agents and infectious viruses can be readily aerosolized and released into the atmosphere, which is a potential threat to the national security [8]. The study of aerosols involves a collective effort from engineering, physics, chemistry, and biochemistry to address existing challenges in various topics such as aerosol instrumentation, light scattering, surface chemistry, bio-variability, formation and loss, etc. [9,10,11]. One of the challenges is characterization and detection of single, airborne particles in their native atmospheric states, i.e., freely suspended in air, without surface interactions with a substrate or particle accumulation in sampling.

Many different techniques have been used for characterization and detection of aerosols. Microscopy techniques such as optical microscopy and scanning electron microscopy are typically used to characterize physical properties, such as size, shape, and structure [12,13]. Mass spectrometry and X-ray spectrometry are used for measurements of elemental compositions [14,15]. Raman spectroscopy (RS), laser-induced fluorescence (LIF), and polymerase chain reaction are typically utilized for analyzing chemical and biological compositions [16,17,18,19]. These techniques require collecting a sufficient volume of samples and then placing the samples on a substrate or flowing into a tube or a vessel for subsequent measurements [20]. In this case, the sample’s properties may be changed in the sampling procedures, and interference may be introduced by interactions between the particle’s surface and its surrounding. Additionally, aerosol particles dispersed in air are in an extremely low concentration; thus, measurement of these aerosol particles often requires the collection of sample particles in a sufficient sample volume [20,21], and this resulting measurement may yield different information than that carried by the individual particles. Furthermore, physical and chemical properties of aerosol particles in air are changing all the time due to change in humidity, temperature, or chemical reaction with atmospheric molecules and air pollutants [22,23,24,25,26,27]. These natural processes in the atmosphere cannot be accurately measured if aerosol particles are sampled using conventional means and measured using a bulk sample.

In recent years, the study of single particles levitated in air has been increasingly reported [28,29,30,31,32,33]. Single-particle studies avoid the interferences caused by the interaction between the particle surface and its surroundings, including substrates and surface modifications during the sample handling [34]. Furthermore, the native state of a particle in the atmosphere can be simulated by levitating the particle in air [35]. On the other hand, compared with using a bulk sample, which can only provide the averaged information of the samples, using a single particle facilitates more precise and specific information about each particle, such as the particle phase, mixing state, and chemical makeup, as well as the variance in the sample [36]. To this end, one way to study aerosol particles is to levitate or trap a single particle in air or in a controlled reactive environment using optical trapping (OT).

Optically levitating a particle was first demonstrated by Ashkin in the 1970s [37]. Over the last several decades, OT and manipulation technologies have been significantly developed [38,39,40,41]. Now, a universal optical trap (UOT) can be applied to trap a single particle of a wide range of size (sub micrometer to ~100 microns), morphology, and materials in air [36,42]. After achieving stable trapping, advanced optical and spectroscopy techniques, such as cavity ringdown spectroscopy (CRDS), elastic light scattering (ELS), RS, etc. can be applied for subsequent measurements. Raman spectroscopy has been used for aerosol characterization and detection due to the high specificity of the structural identification of chemical composition based on vibrational frequencies of molecules. However, conventional Raman spectroscopy also faces the same challenges in the measurement of aerosol particles: the information from averaged measurements using bulk materials and the substrate interference. Furthermore, an additional challenge for Raman spectroscopy is that strong fluorescence can overwhelm a weak Raman signal, especially for bioaerosol particles [43,44]. Nevertheless, the combination of optical trapping and Raman spectroscopy has shown the promise in addressing both challenges.

The first OT-RS study was reported in 1983 by Trunk and Kiefer [45]. In their study, solid glass spheres and nonspherical quartz particles of 10–30 µm size were trapped in air, and the Raman spectra were acquired simultaneously. Recently, OT-RS has been reported for the study of airborne aerosol particles. Hopkins et al. reported that a single aerosol droplet could be controlled by a single-beam gradient-force and characterized by RS in 2004 [46]. Pan et al. reported the first measurements of single-particle Raman spectra for an optically trapped multi-walled carbon nanotube (MWCNT) by photophoretic force in 2012 [47]. Ling et al. (2013) reported on Raman spectroscopy of optically levitated, micron-sized, airborne absorbing particles using a single, focused Gaussian beam [48]. Wang et al. reported the Raman spectra of single pollens and grass spores in air, which were obtained by OT-RS, in 2015 [49]. In that work, five Raman bands were found close to 3000 cm^−1^, where most bands were overwhelmed by the strong fluorescence. Gong et al. reported fluorescence-free Raman spectra of fluorescence-dye-coated spheres optically trapped in air [50]. The results show that the strong fluorescence will be bleached in several seconds. As a result, it was demonstrated that the UOT formed by the counter-propagating hollow beams can effectively reduce or even remove the fluorescence. Later, Gong and coworkers measured Raman spectra using the same OT-RS system from single pollen particles and reported their clean Raman spectra without fluorescence [36]. Very recently, Ai et al. used OT-RS to characterize single, trapped fungal particles in a controlled environment [51]. Single fungal aerosol particles were trapped for a period of time to observe physical and chemical changes over time and to monitor the chemical reaction between a single particle and ozone based on changes in the Raman spectra. More details about recent studies of single, airborne particles using OT-RS can be read in several recent reviews [52,53,54].

In this work, we presented a collection of single-particle Raman spectra using OT-RS. The research objective was to begin development of an open-end data reference of Raman spectral fingerprints of single, airborne particles that have been studied using OT-RS. Such a database is currently not available. This work provides a collection of the single-particle Raman spectra from eight different categories: amino acids, B-vitamins, biowarfare agents, spores, pollens, fungi, extraterrestrial dust, and terrestrial dust, which have been previously published as well as some new data [8,36,49,50,51,55]. As a result, this single-particle spectral database will serve as a useful reference for potential identification and detection of single, aerosol particles in air using OT-RS and for the interpretation of Raman spectra of aerosol particles when exposed to complex conditions including reactive environments. Promise and limitation of the OT-RS technique for characterization, identification, and detection of airborne particles in their native states is also briefly discussed.

## 2. Materials and Methods

### 2.1. OT-RS Setup

**Figure 1:**
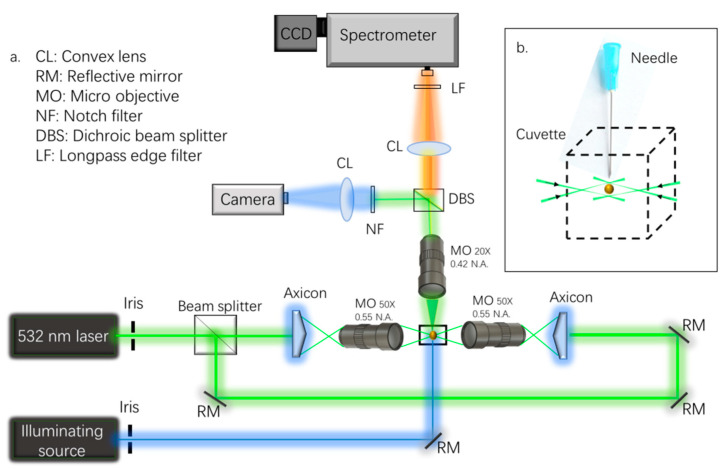
(**a**) The experimental setup of the OT-RS system; (**b**) the schematic shows a particle trapped in UOT.

Figure 1a illustrates the experimental setup of the OT-RS system. A detailed description can be found in previous publications, e.g., [8,25,36,50,51,55]. A continuous wave Gaussian beam at 532 nm (near TEM00 mode) is used for both trapping particles of interest and Raman excitation. The power of the laser is controlled between 50 and 1500 mW to trap a wide variety of single particles. The beam is split into two and converted to two hollow beams using two axicons (cone angle = 170 degree, Thorlabs). The two hollow beams are focused into two optical hollow cones via two micro-objectives (MO) (×50, numerical aperture (N.A.) = 0.55). The optical trapping region is formed between the two foci of the counter-propagating hollow beams, which is termed as the UOT. An octagonal chamber is placed between the two micro-objectives to house the UOT and to facilitate the control of the reactive environment around the trapped particle in the UOT. The size of the focal region is controlled by adjusting the separation distance between two MOs. Figure 1b shows the particles in the form of powder/dust introduced by a syringe needle into the trapping region. The syringe needle is mounted on a translation stage to adjust the position in the x-y plane.

The Raman scattering is excited by the trapping beams and collected by an MO (×20, N.A. = 0.42). Then, the scattering signal passes through a dichroic beam splitter and is divided into two beams. One beam passes a convex lens and a long-pass edge filter, which transmits the Raman scattering to the spectrometer (Acton 2300i, Princeton, NJ, USA). An electron-multiplying charge-coupled device (EMCCD, ProEM, Princeton, NJ, USA) is attached to the Raman spectrometer for data recording. The entrance slit of the spectrometer is set at 100–200 µm.

The grating is set at 600 or 1200 groves/mm. The spectral integration time is varied from 0.01 to 300 s, depending on the signal intensity. The other beam that is split by the dichroic beam splitter passes through a convex lens and is imaged onto a compact CMOS camera that can monitor shape, size, and position of the trapped particle in real time. A collimated laser beam at 405 nm is used to illuminate the trapped particle for imaging. The imaging system is calibrated to 0.18 ± 0.01 µm/pixel while the exposure time of the images is 0.25–0.5 ms and triggered via the EMCCD.

Note that the OT-RS system shown in Figure 1 is a typical OT-RS system that has been used for the studies of airborne particles that are covered in this work. For a specific particle sample, experimental parameters such as trapping laser beam power, size of the UOT, signal integration time, etc. are varied, but within the ranges mentioned above.

### 2.2. Trapping Forces

Trapping airborne particles mainly involves two types of optical forces, photophoretic force (PPF) and the radiation pressure force (RPF) [39,53]. The PPF is created by photopheresis due to differences in the surface thermal accommodation coefficient and in the particle’s surface temperature, which plays a dominant role in trapping strongly absorbing particles. The RPF, which consists of the scattering force and gradient force, can be formed by a tightly focused laser beam to trap non-absorbing particles. Depending on experimental needs, optical traps of various optical configurations have been reported [39,56]. Among them, a UOT that is formed by two counter-propagating hollow beams can trap both absorbing and non-absorbing particles of arbitrary physical and chemical properties. In this work, most samples were trapped using a UOT [8,36,50,51,55]. However, some samples in the previous works were trapped using the photophoretic trapping system, as the particle materials are strongly absorbing. For example, pollen samples are bright and absorb much less light than single-wall carbon nanotube (SWCNT) particles, which are dark. Thus, it is easy to trap SWCNT particles, but hard to trap pollen grains using a photophoretic trapping system. Nevertheless, a UOT is capable of trapping all airborne particles [53].

In Figure 1b, the particle is trapped in a UOT, which is formed by counter-propagating hollow beams. The size of the UOT can be adjusted by precisely tuning the separation distance between the two foci. The separation distance is set between several microns and ~100 microns, depending on particle size and material properties. As illustrated in Figure 1b, the trapping beam does not touch the particle directly. Thus, minimum to no photodamage is induced on the particle surface. But there will be some mass loss caused by the thermal evaporation on the particle’s surface when a thermally sensitive particle is trapped, or the trapping beams’ intensities are too strong. Additionally, the power of the trapping beams and the surface thermal conductivity of a particle are also important factors to determine the trapping time of the particle. In the practice of optical trapping, due to different mass loss rates, some particles can be trapped rigidly for hours or longer, while other particles can be trapped only for a few seconds. Therefore, in order to achieve stable trapping, the trapping beam power must be set in a specific range to minimize the mass loss and maximize the trapping time of the particle. In addition to the surface thermal conductivity, many other factors, such as particle size and morphology, also contribute to the trapping time. Some particles may experience a thermal fragmentation as the particles are hit by the trapping beams when they are introduced into the trapping region. In this case, only one part of a particle is eventually trapped in the UOT. Whether a particle will be fragmentized depends on the particle’s thermal conductivity, size, morphology, power of the trapping beams, and the forces holding the particle together. For instance, strongly absorbing SWCNTs have a high thermal conductivity and can remain unaltered in the UOT. Alternatively, some biological particles, like pollen grains, have lower thermal conductivity and are thermally decomposed easily. When a single bioaerosol particle enters the trapping region, the particle can be fragmentized into sub-micron to micrometer-sized pieces. In such instances, after the fragments settle in the trapping region, one of the fragments will be stably trapped. Even though only one small individual fragment is trapped, the particle-fragment is sufficient to allow the OT-RS system to reveal its chemical composition [35,57].

### 2.3. Sample Materials

Table 1 lists the samples along with their suppliers and a brief description of their properties, such as size, color, morphology, etc. The trapping efficiency denotes how difficult it was to achieve successful trapping. Stability illustrates how long such particles have been stably trapped by the OT system (H: more than one hour, M: 10–60 min, L: below 10 min). There is a strong correlation between trapping efficiency and stability. All samples were in the form of dried powders. Raman spectra were obtained from single, trapped particles. The samples were used as received without further modification or preparation (e.g., coating, heating, etc.).

Aerosol particles have different properties in size, light absorbance, morphology, etc., which offer more flexibility for the particles to be trapped and manipulated. This diversity creates challenges in controlling the particle’s motion and the elaboration of trapping force components. In air, trapping is more challenging than and not as easy as trapping in liquid due to external perturbations such as mechanical vibrations and airflow. However, once a particle is stably trapped, Raman spectra can be measured under experimental control. The samples’ names with their abbreviations used in this paper are all listed in Table 1 as follows: glycine, L-glutamic, L-threonine, Vitamin B5 (B5), Vitamin B7 (B7), Vitamin B12 (B12), Bacillus globigii (BG), Yersinia rhodei (YR), MS2, Bacillus subtilis (BS), Johnson grass smut spore (JG), Bermuda grass spore (BGS), Perennial ryegrass (rye), Western ragweed (ragweed), Paper mulberry (mulberry), and English oak (oak), Aspergillus fumigatus (AF), Aspergillus versicolor (AV), Cladosporium herbarum (CH), Paecilomyces variotii (PV), Penicillium camembertii (PCa), Penicillium chrysogenum (PCh), Penicillium digitatum (PD), lunar-regolith simulant (LS), Mars analog (MA), volcanic ash (VA), carbon sphere (CS), silica microspheres (SM), SWCNT, MWCNT, and rhodamine B doped polyethylene microspheres (RhB-PEMS).

## 3. Results and Discussion

### 3.1. Single-Particle Raman Spectra of Standard Samples (Validation of the OT-RS System)

**Figure 2:**
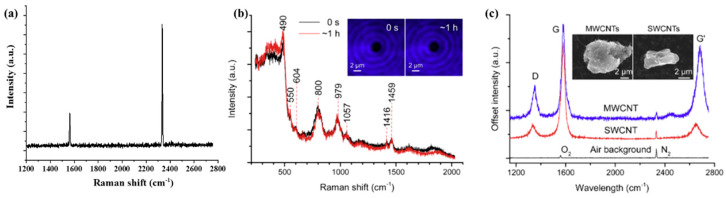
(**a**) Raman spectra of oxygen and nitrogen in ambient lab air, (Reproduced with permission from [8]. Copyright 2021, IOP science). (**b**) Raman spectra of single, trapped SM, (**c**) Raman spectra of single, trapped SWCNT and MWCNT. (Reproduced with permission from ref. [36]. Copyright 2018, Elsevier).

In general, the SWCNT, MWCNT, and SM were used to calibrate the OT-RS system. The entrance slit of the spectrometer was set at 100 μm for the spectra acquisition. A grating with 1200 groves/mm was used, and the integration time was set at 60 s for the data acquisition. The laser power was set at 50 mW to trap SWCNT and MWCNT, and 700 mW to trap SM. The Raman spectra of the ambient lab air are illustrated in Figure 2a [8]. The typical Raman bands of oxygen and nitrogen are located at 1558 cm^−1^ and 2331 cm^−1^, respectively. The spectral resolution calculated by the full width at half maximum of the nitrogen band is ±4 cm^−1^. Figure 2b is the Raman spectra of a single trapped SM [36]. The Raman peaks at 490 cm^−1^ and 605 cm^−1^ present the breathing modes of 4- and 3-membered rings. The peaks at 800 cm^−1^ and 980 cm^−1^ are attributed to the optical mode of the SiO_2_ and (OH)-Si, respectively. As shown in Figure 2b, even after trapping for one hour, there is no spectral change found. Therefore, Figure 2a,b are used for the spectra calibration of the OT-RS system. Figure 2c shows the Raman spectra of the single, trapped SWCNT and MWCNT clusters using OT-RS [36]. The common Raman bands of carbon materials are located at 1349 cm^−1^, 1581 cm^−1^, and 2684 cm^−1^, which correspond to the D-band, G-band, and G’-band, respectively. The signal-to-noise ratio (SNR) can be calculated by SNR = $\bar{S}$/σ_y_ of the CNT spectra, where $\bar{S}$ is the average peak intensity and σ_y_ is the deviation of the peak intensity. The spectrum SNR is larger than 50, which is quite good. Once these validations are complete, the OT-RS system is ready to be used.

### 3.2. Raman Spectral Fingerprints of Single Bioaerosol Particles

#### 3.2.1. Amino Acid

**Figure 3:**
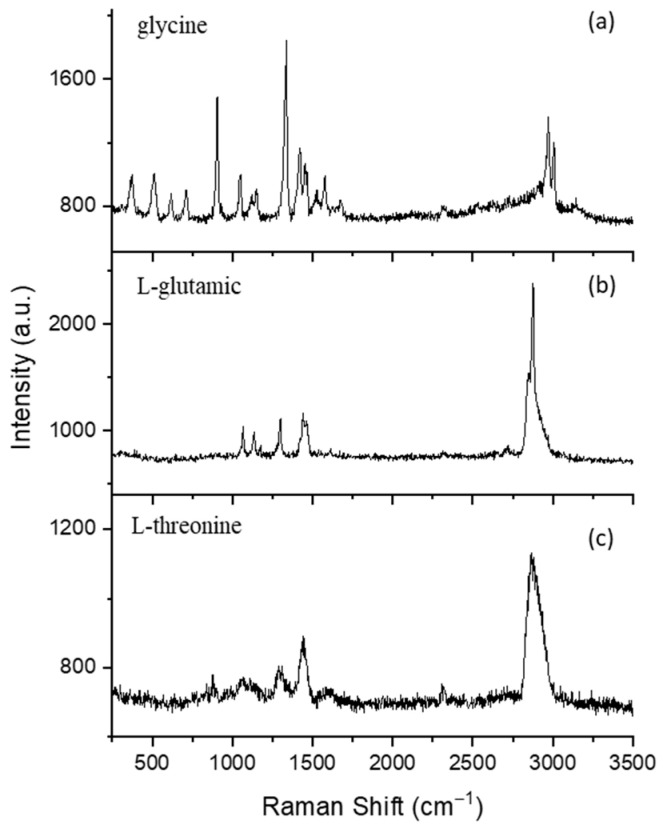
Typical Raman spectra of three types of single amino-acid particles measured by the OT-RS: (**a**) glycine, (**b**) L-glutamic, and (**c**) L-threonine.

Chemical compositions of amino acid were characterized by using the single-particle Raman spectra obtained by the OT-RS system. The entrance slit was set at 100 μm. A grating with 600 groves/mm was used. The integration time was set at 60 s for the data acquisition. The trapping laser power was set between 400 and 700 mW based on the trapping stability of each trapped particle. Figure 3 shows typical Raman spectra of three types of amino acid particles (glycine, L-glutamic, and L-threonine). All three spectra were obtained in the first minute after a particle was stably trapped.

##### Glycine

The spectrum in Figure 3a was obtained from a single glycine particle trapped in air. Most bands appear in the low-wavenumber region. The CH_2_ torsional mode is observed at 369 cm^−1^ [58]. In the zwitterion form, the amino acids exhibit the COO^−^-rocking vibrational mode in the wavenumber region of 500–550 cm^−1^. In this work, the rocking COO^−^ vibrational mode in the glycine molecule is coupled with C-N deformational mode, which was observed at 513 cm^−1^ [58,59,60]. The COO^−^ wagging vibrational mode is observed at 616 cm^−1^ [58]. Generally, the COO^−^ scissoring vibrational modes occur in the region of 650–750 cm^−1^. In the present investigation, the COO^−^ scissoring is coupled with C-C stretching vibration that is obtained at 709 cm^−1^ [58]. Mostly, the rocking CH_2_ vibrational modes occur in the region 750–950 cm^−1^ [58]. In this work, the CH_2_ rocking vibrational mode is coupled with COO^−^ deformational mode that is obtained at 905 cm^−1^, which is the most massive band in the glycine spectrum [58]. Bands at 1048 and 1122 cm^−1^ are assigned to C-NH_2_ stretching and NH_3_^+^ rocking vibrational modes that are coupled with the CH_2_ twisting vibrational mode [58,61]. The wagging vibrational mode of the CH_2_ group generally appears in the region of 1330–1360 cm^−1^. The CH_2_ wagging vibrational mode of glycine molecules is obtained at 1338 cm^−1^ which is the second intense peak in the glycine spectrum [58]. The CH_2_ scissoring in the plane-bending vibrational mode is coupled with the COO^−^ symmetric and asymmetric stretching vibrational mode, which are obtained at 1416 cm^−1^ and 1452 cm^−1^, respectively [58]. The NH_3_^+^ symmetric deformation mode is observed at 1580 cm^−1^ [58,62]. On the other hand, amino acids show weaker Raman bands in the region 1590–1690 cm^−1^ due to the asymmetric deformation mode of the NH_3_^+^, which can be observed in this work as a weak band around 1677 cm^−1^. The Raman band of N_2_ is observed at 2331 cm^−1^ [8]. The CH_2_ symmetric stretching vibrational modes are observed at 2976 cm^−1^ and 3013 cm^−1^, which are the most common bands in biological materials [58]. A weak and broad band at 3162 cm^−1^ has been assigned to NH_3_^+^ asymmetric stretching vibrational mode [58].

##### L-glutamic

The Raman spectrum of a single, trapped L-glutamic acid particle is shown in Figure 3b. The C-C stretching was observed at 1064 cm^−1^ [63]. A vibration associated with deformation of NH_3_^+^ was observed at 1122 cm^−1^ [60]. The CH_2_ wagging band was obtained at 1301 cm^−1^ [63]. The band at 1443 cm^−1^ has a broad width, which is assigned to the COO^−^ symmetric stretching vibration [64]. The most intense band is in the high-wavenumber region of the spectrum, which corresponds to CH_2_ stretching at 2848 and 2880 cm^−1^ [65].

##### L-threonine

Typical Raman spectrum of a single, trapped L-threonine acid particle is shown in Figure 3c. Threonine is a secondary alcohol group, in which most of its bands are attributed to the C-C stretching vibration or carboxylate group. A vibration that is associated with the CCN stretching mode is observed at 876 cm^−1^ [66]. The peak at 1076 cm^−1^ is assigned to OH deformation motion [66]. The methine group, which is associated with CH_2_ wagging vibration mode, can be seen at 1299 cm^−1^ [67]. A COO^−^ symmetric stretching vibration is observed at 1443 cm^−1^ [60,67]. A strong intensity band associated with the CH_2_ stretching mode is observed 2878 cm^−1^ [67].

Because of the long spectra-acquisition time and no surface interference of a trapped particle, the OT-RS system results in high SNR. For instance, the glycine spectrum in Figure 3a has a smooth baseline compared with spectra collected using a substrate [66]. Furthermore, some interference caused by fluorescence can be observed on the baseline. This is mainly because of the photo-bleach process of UOT. More details about photo-bleaching are discussed in Section 3.2.2 and Section 3.2.3.

In general, the Raman spectra of amino acids are quite different from each other, but there are still some common bands among them. For instance, all amino acids have a carboxyl group (COOH) at one terminus and an amine group (NH_2_) at the other side. They are different in their side chains, which gives amino acids their characteristic chemical properties. In all spectra, the C-C skeletal stretching is observed around the 1000 cm^−1^, and the CH stretching vibration mode is found around 3000 cm^−1^. These are also the common bands for most bioaerosols, as the amino acids are components of proteins and substances for carbon metabolism in most bioaerosols [68]. Thus, these amino-acid spectra can be used for interpretation of the Raman spectra of biological materials.

#### 3.2.2. B Vitamins

**Figure 4:**
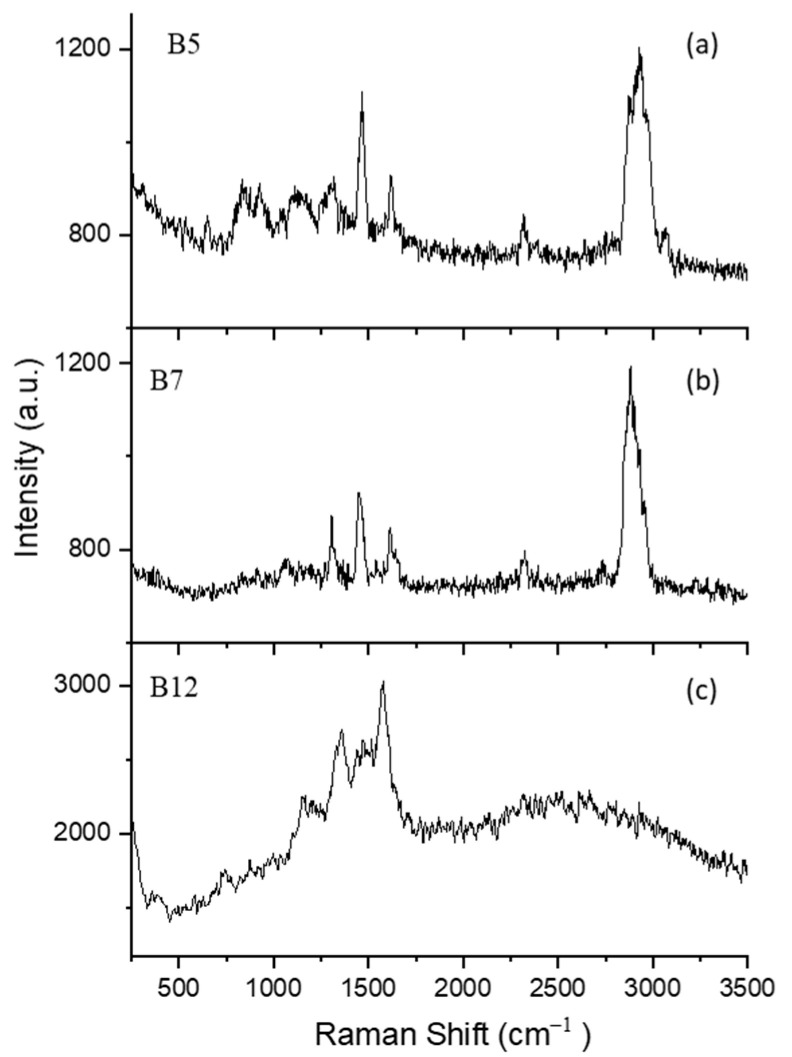
Raman spectra of three types of single, trapped B-vitamins (**a**) B5, (**b**) B7, and (**c**) B12.

Figure 4 shows typical single-particle Raman spectra for vitamins B5, B7, and B12. The experimental parameters were the same as those described in Section 3.2.1. The three spectra were obtained in the first minute after a particle was stably trapped.

##### B5 (D-Pantothenic Acid Hemicalcium Salt)

The spectrum in Figure 4a was obtained from a single B5 particle trapped in air. In the spectrum of B5, the C=C=C angle deformation mode and the C=C stretching vibration mode are found at 658 cm^−1^ and 842 cm^−1^, respectively [69]. In addition, the peak at 933 cm^−1^ belongs to the rocking mode of CH_2_ [69]. Two stretching normal modes attributed to the C-O(H) group are observed at 1041 cm^−1^ and 1139 cm^−1^ [69]. Furthermore, the CH group has a deformation mode at 1306 cm^−1^ [69]. The most intense band at 1477 cm^−1^ is assigned the antisymmetric deformation of the CH_3_ mode and the band at 1628 cm^−1^ is assigned the C=O stretching vibrational mode [69]. The wide wave package around 3000 cm^−1^ is due to the symmetric stretching modes of the CH group.

##### B7 (Biotin)

The observed Raman spectrum of a single B7 particle is shown in Figure 4b. The peak at 1071 cm^−1^ is due to the stretching mode of the ureido ring [70]. The CH and CH_2_ deformation modes are observed at 1318 cm^−1^ and 1458 cm^−1^, respectively [70]. The peak at 1654 cm^−1^ is assigned the C=O stretching vibrational mode [70]. The strong intense peak at 2892 cm^−1^ corresponds to the CH stretching vibration [71].

##### B12 (Cyanocobalamin)

Figure 4c shows the Raman spectrum of a single B12 particle. The weak band at 733 cm^−1^ is assigned the CH_3_ rocking vibration mode [72], and bands between 1149 to 1574 cm^−1^ are attributed to the corrin ring vibration mode [73,74]. The corrin ring is a characteristic structure for B12, which is considered the core of the molecular structure.

Although the three samples shown in Figure 4 are from a vitamin family, they show quite different chemical structures as different Raman bands appear. The special components of ureido and the coring ring for B7 and B12 make their spectra easily differentiated; however, they still share some common Raman bands. The peak around 1300 cm^−1^ corresponds to the CH stretching mode, which is observed in B5 at 1306 cm^−1^ and in B7 at 1318 cm^−1^. In addition, the peak around 3000 cm^−1^ of the CH stretching vibration is observed in B5 and B7. B12 is a highly fluorescent sample, and it is difficult to completely get rid of the fluorescence. Ibáñez et al. reported the Raman spectrum of B12 using surface-enhanced Raman spectroscopy with a 785 nm exciting laser [74]. The results show some Raman bands in the spectrum, but the interference caused by fluorescence is still high. In Figure 4c, some peaks are resolved, but the baseline is still noisy, and many peaks are comparable with the noise, which is mainly caused by the fluorescence of the sample. In this work, the fluorescence may be excited by the trapping laser beam, as a laser beam at 532 nm can induce strong fluorescence in biomaterials, the UOT can reduce the fluorescence through photo-bleach process. In practice, many factors determine the photo-bleach efficiency, such as the fluorescent properties of the sample materials, the laser illumination time, the laser wavelength and intensity, the location of a trapped particle in the UOT, etc. Although the UOT is efficient for single-particle fluorescence bleaching, it is not effective for all sample materials. Different approaches are also being explored to quench the fluorescence; for example, an additional laser beam at a different wavelength may be added to the OT-RS system to illuminate the particle and help further reduce fluorescence.

#### 3.2.3. Pollens and Spores

**Figure 5:**
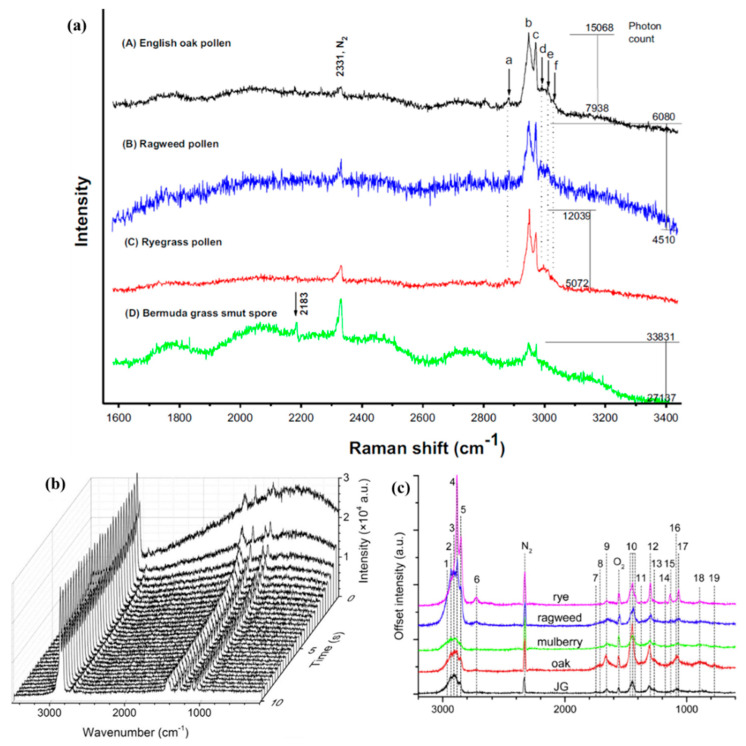
(**a**) Raman spectra of single, trapped spores. (Reproduced with permission from ref. [49]. Copyright 2015, Elsevier). (**b**) Raman spectra of trapped RhB-PEMS. (Reproduced with permission from ref. [50]. Copyright 2017, Elsevier). (**c**) Raman spectra of various types of single, trapped pollen and spore. (Reproduced with permission from ref. [36]. Copyright 2018, Elsevier).

The first Raman spectra of single pollens and fungal spores optically trapped in air were reported by Wang et al. in 2015 [49]. Three pollens of English oak, ragweed, and ryegrass, and a Bermuda grass spore were studied. The single-particle Raman spectra of these four samples were recorded in the spectral range of 1600–3400 cm^−1^. As shown in Figure 5a, five Raman bands were obtained around 3000 cm^−1^, which were assigned to the CH_2_ symmetric stretch (2948 cm^−1^), CH_2_ Fermi resonance stretch (2970 cm^−1^), CH_3_ symmetric stretch (2990 cm^−1^), CH_3_ out-of-plane end asymmetric stretch (3010 cm^−1^), and unsaturated =CH stretch (3028 cm^−1^). Even though the five Raman bands around 3000 cm^−1^ are resolved for the three pollen species and two bands for the Bermuda grass spores, some bands are barely distinctive due to the strong fluorescence background and the baseline noise. Furthermore, no characteristic Raman bands for the samples were found in the hump-like region between 1600 and 2900 cm^−1^. In 2017, Gong et al. reported time-resolved Raman spectra of a single dye-doped polymer particle trapped by a UOT in air [50]. The time-evolution of Raman spectra of the single, dye-doped polymer particle is illustrated in Figure 5b. As the fluorescent particle is trapped in the UOT, the fluorescence can be rapidly photo-bleached in the first several seconds. While this occurs, the baseline flattens, and the clear Raman bands become apparent. Later, the fluorescence-free Raman spectra of pollens and spores (Perennial ryegrass, Western ragweed, English oak, and Johnson grass smut spore) were reported by Gong et al. by using the same OT-RS system [36]. In this experiment, the entrance slit was set at 200 μm, and the integration time was set at 300 s. This larger slit width and longer acquisition time were used to obtain a better SNR signal for each trapped particle. As shown in Figure 5c, the baseline of the Raman spectra is clean, only with minimum influence from fluorescence. Compared with the previous study [49], more information can be revealed by the cleaner Raman spectral fingerprints. For example, the Raman bands of the CH stretching around 3000 cm^−1^ are much better resolved, showing more accurate band positions. Furthermore, many characteristic Raman bands were found in the wavenumber region of 700–1800 cm^−1^. For instance, the C-C stretching mode around 1080 cm^−1^, CH_2_ bending mode at 1450 cm^−1^, and Amide I mode at 1660 cm^−1^, which were not shown in the previous study, were observed in these fluorescence-free Raman spectra.

In general, many organic functional groups contain fluorophores, which may generate strong fluorescence in the detected Raman shift region, depending on the excitation wavelength. For some biological samples, fluorescence can be generated by a laser excitation wavelength from near UV to near IR. In this work, as the Raman signal was excited directly by the trapping beam wavelength at 532 nm, strong fluorescence can be generated. As optical trapping is less sensitive to trapping laser wavelength thought PPF is based on light absorbance that is wavelength dependent, we may use different trapping laser sources to avoid strong fluorescence interference for different sample materials. It is challenging to completely eliminate the fluorescence in the Raman spectra, regardless of what methods or techniques are used. Photo-bleaching is an efficient way to quench the fluorescence. The conventional method is long-time irradiation of the samples that are placed on a substrate, using mercury lamps, ultraviolet, or a visible laser. The irradiation time can range from minutes to several hours [49]. This approach is not only time-consuming but also may damage the sample’s surface due to the uneven heating and the slow heat dissipation. Comparatively, the photo-bleaching process from the UOT is fast and without any photo-induced damage. An optically levitated particle in the UOT is engulfed by the scattered light from the trapping beams and the entire surface of the particle is photo-bleached. In this case, even though a high laser power is used, the local temperature induced resulting from laser heating is still much lower than when the particle is placed on a substrate. In addition, the photo-bleaching process can be completed in several seconds because the particle is trapped inside the UOT where photons are coupled and pass throughout the whole particle through scattering and reflections, and the resonance effect of whispering gallery modes (WGM) can further enhance the bleaching effect if the particle is spherical [50]. As a result, in most cases, the OT-RS can effectively reduce the fluorescence by a fast photo-bleaching process.

#### 3.2.4. Aerosolized Biowarfare Agent (BWA) Surrogates

**Figure 6:**
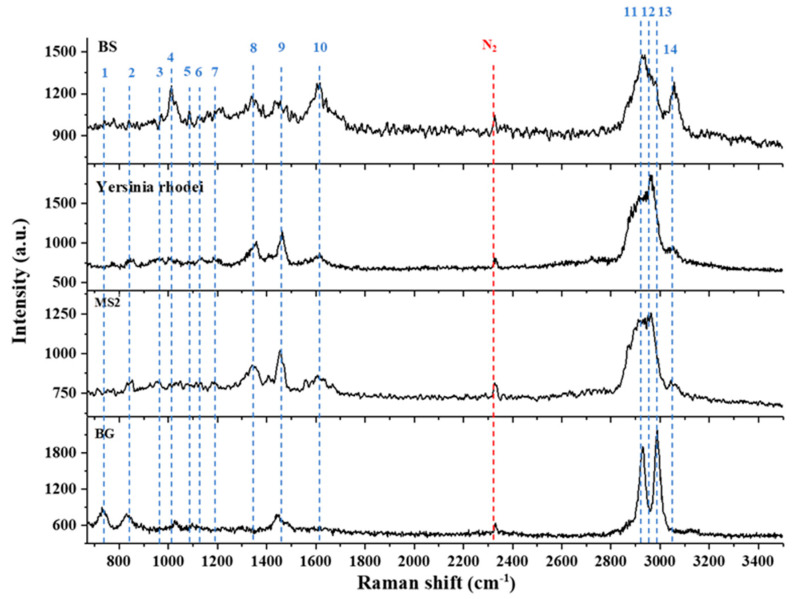
Raman spectra of BWA surrogates. (Reproduced with permission from ref. [8]. Copyright 2021, IOP science).

In a previous work, BWA surrogates were studied using OT-RS [8]. In this experiment, the slit width of the spectrometer was set at 200 μm, and the integration time of 60 s was used for the spectra acquisition. The single-particle Raman spectra of four different types of BWA surrogates (BG, YR, MS2, and BS) were collected for species classification and potential identification. Due to the photo-bleaching process, the Raman spectra of the four BWA surrogates shown in Figure 6 are clean, with low baseline noise generated by fluorescence. As shown in Figure 6, the spectra have some similarities; for example, the wave package around 3000 cm^−1^ was seen in all the samples. These common bands are related to CH stretching that relates to lipids or proteins in most biomaterials as discussed in the previous section. However, even though some common bands are found in this region, the spectral structures are different and can be used to classify the different samples. Furthermore, the Raman spectra in Figure 6 also revealed distinctive bands for each sample. For example, the Raman band at 732 cm^−1^ was only seen in the spectra of BG; the band at 955 cm^−1^ which comes from the C-C backbone appears only in the spectra of BS and MS2; the band at 1032 cm^−1^ that is from phenylalanine was unique for BS among the four samples. Therefore, different BWA surrogates can be identified by these characteristic spectral fingerprints. The principal component analysis for the four BWA surrogates as well as three common atmospheric bioaerosols (JG, oak, and ragweed) further demonstrated that the four BWA surrogates can be identified, and all the samples can be clearly classified by their single-particle Raman spectra [8].

#### 3.2.5. Fungal Particles

**Figure 7:**
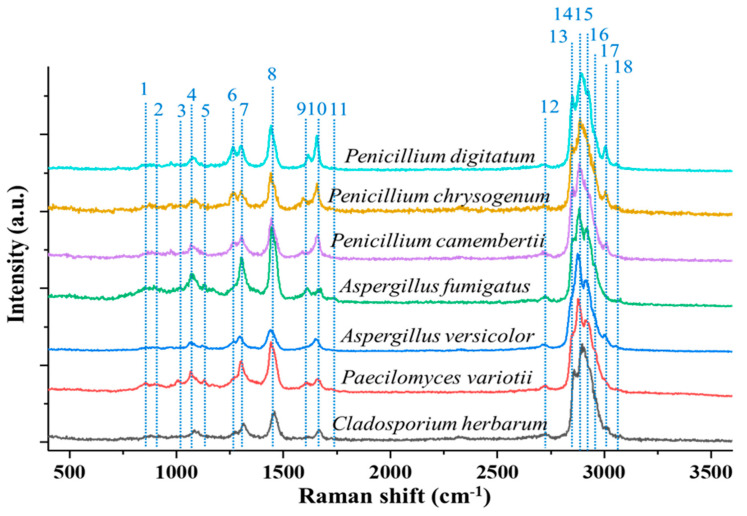
Raman spectra of various types of single, trapped fungi. (Reproduced with permission from ref. [51]. Copyright 2022, the Royal Society of Chemistry).

Recently, the Raman spectra of single, fungal aerosol particles were measured using OT-RS by Ai et al. [51]. In this experiment, the trapping laser power was set at 1500 mW to obtain the maximum stability for the particle trapping. Similar to the previous section, the slit width of the spectrometer was set to 200 μm, and the integration time of 60 s was used for the spectra acquisition. Seven different fungus samples were used: Aspergillus fumigatus, Aspergillus versicolor, Cladosporium herbarum, Paecilomyces variotii, Penicillium, Camembertii, Penicillium chrysogenum, and Penicillium digitatum. The single-particle Raman spectra of these samples are shown in Figure 7. All the fungus samples share the most common Raman bands of bioaerosols, such as the C-C stretching around 1000 cm^−1^ and CH stretching band packages around 3000 cm^−1^. In addition, some distinguishable bands which may reveal specific properties of each fungus also can be seen. For example, the Raman band at 1131 cm^−1^ arising from palmitic acid and the band at 1750 cm^−1^ from lipids or C=O were found in the Aspergillus species only. The band around 1267 cm^−1^ associated with the CH stretching (lipids in normal tissue) was seen only in the Penicillium type of fungus. These Raman bands are indicative of the distinctive chemical composition or properties of the specific fungi or fungal species; therefore, different fungal species can be classified by these characteristic spectral fingerprints.

In addition to the characteristic spectral features that are obtained when single particles are trapped in air, the single-particle Raman spectra also can be used to study surface chemistry when a trapped particle is exposed to a reactive environment, such as ozone [51]. The time-resolved, single-particle Raman spectra show a uniform decrease of the peak intensity with time when a particle is trapped in air. This is mainly because of the mass loss that is caused by the surface chemical evaporation. When trapped in ozone, Raman bands showed nonuniform changes in terms of their peak intensities, reflective of the chemical changes occurring. For example, a decrease of intensity of the band at 840 cm^−1^ is caused by the reaction of tyrosine with O_3_. The increase of the Raman band intensity at 1005 cm^−1^ is due to the lipid oxidation. In such cases, the OT-RS technique is capable of monitoring the chemical reactions based on the spectral features of the bands. Consequently, Raman spectral fingerprints from OT-RS can not only be used to identify and classify aerosol particles when exposed in air, but also be used for the study of chemical reactions in the simulated native state.

### 3.3. Raman Spectral Fingerprints of Extraterrestrial and Terrestrial Dust Simulants

**Figure 8:**
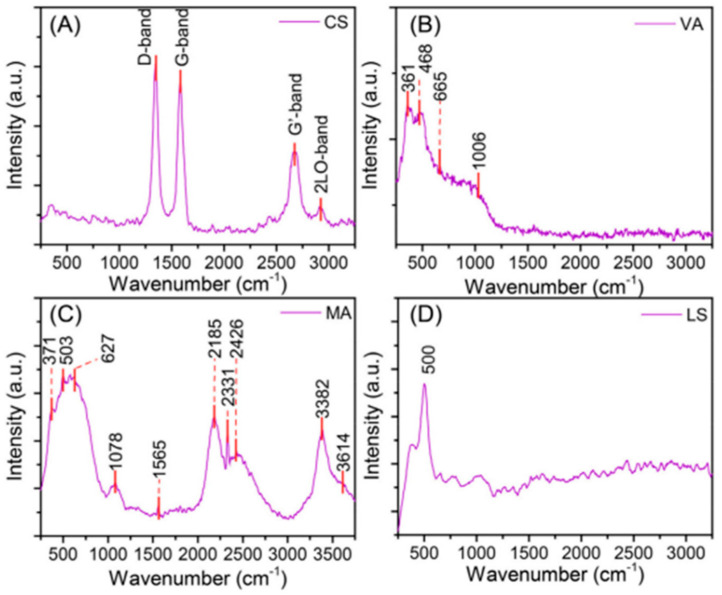
Raman spectra of a single, trapped (**A**) carbon sphere, (**B**) volcanic ash, (**C**) Mars analog, and (**D**) lunar-regolith simulant. (Reproduced with permission from [55]. Copyright 2020, Elsevier).

The Earth’s atmosphere contains interplanetary dust particles, which play an important role in the Earth’s ecosystem and may also carry the primitive information of the early Solar system. Very recently, OT-RS has demonstrated itself to be a powerful tool for the study of extraterrestrial dusts particles [55]. In this experiment, the entrance slit was set at 200 μm. A grating with 600 groves/mm was used, and the integration time was set at 60 s for the data acquisition. Figure 8 shows single-particle Raman spectra obtained by OT-RS from two extraterrestrial dust simulants that include a lunar-regolith simulant and a Mars analog, and two terrestrial dust simulants that are a carbon sphere and volcanic ash.

The Raman spectrum shown in Figure 8A was acquired from a trapped single carbon sphere. The common bands of carbon materials were found at 1367 cm^−1^ (D-band), 1585 cm^−1^ (G-band), and 2700 cm^−1^ (G’-band). In addition, one more band was found at 2900 cm^−1^, which corresponds to the 2LO-band. Those band features can be used to distinguish structural and compositional characteristics of different carbonaceous materials. Figure 8B shows the single-particle Raman spectrum of volcanic ash. The main chemical compositions of volcanic ashes are SiO_2_ glasses, ferric minerals, and sulfate. The Raman band at 361 cm^−1^ is from the Ca-O stretching vibration. The band at 468 cm^−1^ is assigned to SiO_2_. The band around 665 cm^−1^ is related to silicate minerals, which is assigned to the Si-O-Si symmetrical band. The peak at 1006 cm^−1^ is attributed to band of sulfates ${\mathrm{SO}}_{4}^{2-}$ in calcium sulfate. Figure 8C,D show the single-particle Raman spectra of extraterrestrial dust simulants. The Raman spectrum of a single Mars analog particle is shown in Figure 8C. The Raman band at 371 cm^−1^ is attributed to the Mg-O stretching of pyroxenes and the peak at 503 cm^−1^ is the O-Si-O band of pyroxenes. Another band around 627 cm^−1^ relates to the bending of Si-O-Si, which comes from end-member Mg-Fe-Ca pyroxenes. These bands indicate that the major composition of this Mars analog is pyroxenes. In addition, the Raman band at 1565 cm^−1^ is from the G-band, which indicates the existence of amorphous or graphitic carbon. Figure 8D shows the Raman spectrum of a single lunar-regolith particle. Principle materials in lunar regolith are pyroxene and plagioclase, along with ilmenite and olivine. The Raman band at 500 cm^−1^ refers to the plagioclase group, which is associated with the symmetric T-O-T stretching mode of the four member TO_4_ tetrahedral ring, where T stands for Al or Si.

Table 2 summarizes the spectral characteristics of all single particles measured using OT-RS to date. More than 100 chemical function groups from single trapped particles in air or in a reactive environment have been assigned based on references of the Raman spectra measured using bulk samples. Note that a Raman band or a Raman shift (cm^−1^) from the same function group, e.g., the CH stretching mode, can be slightly different in individual molecules containing the same function group, or same molecules from individual aerosol particles. One spectral feature (band location, intensity ratio, band shape, absence and presence of a bands, etc.) may not be used for identification of a chemical composition; however, a combination of such features of several function groups can be a unique identification of a specific chemical constituent or a specific type of aerosol particles [8,25,51]. Given a database of such features, advanced data-analysis methods such as principal component analysis [8] and machine learning [75] can be utilized for spectral pattern formation and recognition to achieve particle identification. Note that some spectral data on single particles using other particle levitation techniques such as electrodynamic balance are not included here. Molecular fingerings from single particles in future studies are strongly encouraged to be added to this open-end collection.

## 4. Future OT-RS for Potential Identification and Detection of Single Airborne Particles

The OT-RS technique enables us to characterize single particles in air or under a controlled reactive environment without the need for using a substrate or a sample container [8,25,36,49,50,51,55]. The technology is advantageous in aerosol characterization in the following aspects:

It reveals a particle’s chemical composition and molecular structure based on spectral features. Single-particle Raman spectroscopy can reveal both the average information of bulk materials and specific information of individual particles.

1. It helps minimize particle surface contamination, modification, and interaction with its surroundings.
2. It helps mitigate background fluorescence interference via photo-bleaching and the WGM resonance effect [50].
3. Measured data reveal temporal information about a particle in a simulated atmospheric state while the particle is being measured.
4. It has high temporal and spatial resolutions (µs, and 300 nm × 300 nm), which facilitate the study of surface properties of a particle and its time-evolution.
5. Integrated with an imaging system, it can concurrently monitor changes in physical and chemical properties of single particles in near-real time.
6. It requires minimal volume of samples, essentially a single particle, which is critically important in detecting or sensing airborne particles in low concentration, e.g., life-threatening viruses and aerosolized BWA particles spread in air.

However, in terms of identification and detection of airborne particles using the OT-RS technique, there are several challenges to be met and further developments are expected in future research:

1. A large, comprehensive database of Raman spectral features is needed. Although Raman spectra of most small molecules or chemical function groups have been established to date, the spectral data have been obtained predominantly using bulk samples and such samples are placed on a substrate. As a result, the specificity of Raman spectral features (relative peak intensity patterns, band structures, spectral overlap, etc.) for individual airborne particles is low and the fluorescence interference may hinder Raman-band assignments. For example, the formation and loss single airborne aerosol particles, which cannot be found by the averaged information from the Raman spectra from bulk measurements, can be characterized by the single-particle Raman spectra via observation of specific or signature Raman band(s) or the relative peak intensity patterns of the particle in each growth/loss phase [43]. On the other hand, in the atmosphere, the surface of each aerosol particle is chemically heterogeneous. Chemical reagents resulting from air pollution process (e.g., contamination) and the solar/UV radiation (e.g., photolytic reactions) can aggressively change the properties of the particle’s surface [51]. These changes cannot be characterized using measurements from bulk materials because the changes can be highly time and particle dependent and can be easily overwhelmed by the averaged Raman signal from the bulk materials. Finally, for some bioaerosol particles that usually contain some fluorescent compounds, the OT-RS technique can effectively mitigate the background fluorescence interference via the efficient photobleaching effect from the optical trap [50]. This point fundamentally differentiates the OT-RS from the bulk-sample RS. Therefore, the existing Raman spectra database cannot replace the single-particle Raman spectral database. Although the collection of single, airborne particle spectral features is not included in this work, only a limited number of particle types have been studied to date. Further collective efforts are needed to expand this data to include the vast number of aerosol types in existence.
2. Given a sufficient database of single-particle Raman spectral features available for single-particle identification, it is necessary to develop data-processing algorithms to link measured spectral patterns to specific airborne particle or its chemical composition. Such studies are rare.
3. Current studies of single particles using OT-RS are lab-based. In real-world applications, the characterization of single particles will rely on deployable, rugged, easy-to-use instruments. Development of such instrumentation is only beginning and must address several individual challenges such as automated, single-particle sample introduction, trapping efficiency and rigidness in the presence of field perturbations (airflow, vibration, etc.), as well as cost-effectiveness. Experience and knowledge derived from existing aerosol instrumentation can be helpful, yet new instrumentation schemes and innovative designs are expected in future studies.
4. Like the evolution of many other technology developments, we should keep our minds open. This includes integrating OT-RS with other advanced optical and spectroscopic techniques (e.g., CRDS, LIF, ELS), as well as new data-processing algorithms (e.g., machine learning) to enhance detection specificity.

## 5. Summary

Raman spectroscopy is based on chemical identification and detection at the molecular level. Recent developments in single, airborne particle handling using optical trapping and manipulations allow for the integration of optical trapping with Raman spectroscopy, and the OT-RS technique has demonstrated great promise in single-particle characterization, identification, and detection of atmospheric aerosols. To this end, one of our knowledge gaps is that we lack a single-particle Raman spectral database, namely, Raman spectra measured from single particles freely suspended in air or under controlled environments. As recent developments of the OT-RS technology advance rapidly, expanded applications of OT-RS in lab-studies and OT-RS-based instrumentation for future field work are highly conceivable. Therefore, there is a need to build a reference for single-particle, Raman spectra. In this work, we presented a new collection of Raman spectra of single-particles from eight different chemical groups (amino acid and B- vitamin, BWA, pollens, fungi, spores, extraterrestrial dust, and terrestrial dust) using OT-RS. This work represents a first step toward establishing a spectral database for single, airborne particles, as a starting point, a complete data bank requires the collection of OT-RS from continued additions of more single, airborne particles of any kind [76]. Parallel to ongoing engineering efforts in OT-RS instrumentation, this open-end database may serve as a valued reference for future identification and detection of single airborne particles using the OT-RS technique. In this work, advantages of OT-RS in single-particle studies and challenges in identification and detection using OT-RS in future studies were also discussed.

## Figures and Tables

**Table 1:** A list of the samples along with their suppliers and brief descriptions.

Sample Class	Name ^(a)^	Supplier	Descriptions (Size, Shape, Color, etc.)	Trapping Efficiency ^(b)^	Stability	Ref.
Standard samples	SWCNT	US nano-Materials (Houston, TX, USA)	Cylinderical shape, black	H	H	[36]
	MWCNT			H	H	
	SM	White House Scientific (Chester, PB, USA)	2 µm, silica shpere, white	L	H	
Amino Acid	Glycine L-glutamic L-threonine	Sigma-Aldrich (St. Louis, MO, USA)	Submicron to several microns,	H	H	
			irregular shape, white to off-white	M	H	
				H	H	
Vitamin	B5	Sigma-Aldrich	Submicron to several microns, irregular shape, white to off-white, except for B12 is glitter dark red	L	M	
	B7			L	M	
	B12			M	H	
BWA	YR	Edgewood Chemical Biological center (Seattle, WA, USA)	~10 µm, irregular shape, light brow	M	M	[8]
	MS2			M	M	
	BS			M	M	
	BG			H	M	
Pollens	rye	Greer lab (Lenoir, NC, USA)	~35 μm, irregular shape, light brown	M	M	[36,49]
	ragweed		~24 μm, irregular shape, yellow	L	M	
	mulberry		~14 μm, irregular shape, light brown	L	M	
	oak		~34 μm, irregular shape, yellow	M	M	
spores	JG	Greer lab	~8 μm, irregular shape, dark brown	L	L	[36,49]
	BGS		5.6–8.2 μm, irregular shape, dark black	M	L	
Fungi	AF	Greer lab	Actual size is in millimeters, the	M	M	[51]
	AV		samples were further ground	M	L	
	CH		to submicron to tens of	M	M	
	PV		microns for experimental	M	M	
	PCa		purpose. Irregular shape,	M	M	
	PCh		black and dark gray	L	L	
	PD			L	L	
Extraterrestrial dust	LA	Johnson sapce center (Houston, TX, USA)	~4 µm, irregular shape with clear edge	L	M	[55]
	MA		Irregular shape, more	L	M	
			stickie, brown			
Terrestrial dust	VA	volcanic eruption	Irregular shape, dark gray	M	H	[55]
	CS	Sigma-Aldrich	2–4 μm, spherical, black	H	H	
Fluorescent particle	RhB-PEMS	Cospheric (Santa Barbara, CA, USA)	10–30 μm, sphere RhB% = ~1%	H	H	[50]

^(a)^ Abbreviations of the names used. ^(b)^ The trapping efficiency in terms of H (high), M (moderate), and L (low).

**Table 2:** A collection of Raman spectral fingerprints from single particles optically trapped in air.

Class	Name	RS Bands (cm^−1^)	Assignments	Ref.
Amino Acid	Glycine	369	CH_2_ torsional mode	[58]
		513	COO^−^ coupled with C-N deformation mode	[58,59,60]
		616	COO^−^ wagging	[58]
		709	COO^−^ coupled with C-C stretching	[58]
		905	CH_2_ rocking coupled with COO^−^ deformation	[58]
		1048	C-NH_2_ stretching coupled with CH_2_ twisting	[58,61]
		1122	NH_3_^+^ rocking coupled with CH_2_ twisting	[58,61]
		1338	CH_2_ wagging vibration	[58]
		1416	CH_2_ scissoring coupled with COO^−^ symmetric stretching vibration	[58]
		1452	CH_2_ scissoring coupled with COO^−^ asymmetric stretching vibration	[58]
		1580	NH_3_^+^ symmetric deformation mode	[58,62]
		1677	NH_3_^+^ asymmetric deformation mode	[58]
		2331	N_2_	[8]
		2976	CH_2_ symmetric starching vibration	[58]
		3013	CH_2_ symmetric starching vibration	[58]
		3162	NH_3_^+^ asymmetric stretching vibration	[58]
	L-glutamic	1064	C-C stretching vibration	[63]
		1122	Deformation of NH_3_^+^	[60]
		1301	CH_2_ wagging band	[63]
		1443	COO^−^ symmetric stretching vibration	[64]
		2848	CH_2_ stretching	[65]
		2880	CH_2_ stretching	[65]
	L-threonine	876	CCN stretching	[66]
		1076	OH deformation motion	[66]
		1299	CH_2_ wagging vibration	[67]
		1443	COO^−^ symmetric	[60,67]
		2878	CH_2_ stretching	[67]
Vitamin	B5	658	C=C=C angle deformation mode	[69]
		842	C=C stretching	[69]
		933	CH_2_ rocking	[69]
		1041	C-O(H) stretching	[69]
		1139	C-O(H) stretching	[69]
		1306	CH deformation	[69]
		1477	CH_3_ antisymmetric deformation	[69]
		1628	C=O stretching	[69]
		3000	CH stretching	[69]
	B7	1071	Ureido ring	[70]
		1318	CH deformation	[70]
		1458	CH_2_ deformation	[70]
		1654	C=O stretching vibration	[70]
		2892	CH stretching vibration	[71]
	B12	733	CH_3_ rocking vibration	[72]
		1149	Corrin ring vibration	[73,74]
		1359	Corrin ring vibration	[73,74]
		1459	Corrin ring vibration	[73,74]
		1574	Corrin ring vibration	[73,74]
BWA surrogates	YR	732	C-C adenosine ring stretching	[8]
	MS2	840	Tyrosine	[8]
	BS	955	C-C backbone stretching, protein	[8]
	BG	1032	Phenylalanine	[8]
		1080	C-C stretching, C-O-C glycosidic link	[8]
		1136	C-N or C-C stretching	[8]
		1187	Cytosine, nucleotides: base ν(CN), guanine	[8]
		1350	Amide III	[8]
		1451	Lipids, protein, or the C-H_2_ deformation	[8]
		1618	Amide I	[8]
		2720	CH stretching	[8]
		3100	CH stretching	[8]
Pollens and spores	rye	771	ν(O-P-O) diester, phosphatidylinositol	[36,49]
	regweed	892	C-C skeletal stretching, C-O-C backbone	[36,49]
	mulberry	1065	C-C or C-N stretching	[36,49]
	oak	1080	C-C skeletal stretching mode, C-O-C glycosidic link	[36,49]
	JG	1136	C-C or C-N starching	[36,49]
	BGS	1177	Cytosine, nucleotides: base ν(CN), guanine	[36,49]
		1265	Proteins, Amide III	[36,49]
		1298	CH_2_ twisting, fatty acid, lipids	[36,49]
		1420–1480	Lipids, protein, CH_2_ deformation	[36,49]
		1660	Amide I, lipids, v(C=C)	[36,49]
		1713	C=O	[36,49]
		1750	Lipids, C=O	[36,49]
		2720–3000	CH stretching, sporopollenin	[36,49]
Fungal aerosols	AF	840	Tyrosine	[51]
	AV	892	C-O-C backbone, C-C skeletal stretches	[51]
	CH	1032	Phenylalanine	[51]
	PV	1080	C-O-C glycosidic link, C–C skeletal stretches	[51]
	PCa	1131	Palmitic acid	[51]
	PCh	1267	Triacylglycerol, CH (lipid in normal tissue)	[51]
	PD	1298	CH_2_ deformation	[51]
		1450	Lipid/protein, CH deformation	[51]
		1610	Cytosine	[51]
		1660	Amide I	[51]
		1750	Lipids, C=O	[51]
		2739	Stretching vibrations of CH	[51]
		2855	CH_2_ symmetric stretch of lipids	[51]
		2878	CH_2_ symmetric stretch of lipids	[51]
		2915	CH_2_ stretch lipids and proteins	[51]
		2960	Out-of-plane chain end antisymmetric CH_3_ stretch band	[51]
		3008	νas(=CH), lipids, fatty acids	[51]
		3059	(C=CH) aromatic stretching	[51]
Extraterrestrial dust	LA	500	Symmetric T-O-T stretching mode (T = Al or Si)	[55]
	MA	371	Mg-O stretch	[55]
		503	O-Si-O band	[55]
		627	Si-O stretching vibrations	[55]
		1078	Band of the ${\mathrm{Co}}_{3}^{2-}$ ion	[55]
		1565	G-band of graphite	[55]
		3382	O-H	[55]
		3614	O-H	[55]
Terrestrial dust	VA	361	Ca-O stretching vibration	[55]
		468	Quartz SiO_2_	[55]
		665	Si-O-Si symmetric band	[55]
		1006	Sulfates ${\mathrm{SO}}_{4}^{2-}$	[55]
	CS	1367	D-band of graphite	[55]
		1585	G-band of graphite	[55]
		2700	G’-band	[55]
		2900	2LO-band	[55]
	SWCNT	490	Breathing modes of 4- and membered rings	[36]
	MWCNT	604	Breathing modes of 4- and membered rings	[36]
		800	Optical mode of the SiO_2_ network	[36]
		979	Vibration of (OH)-Si	[36]
		1349	D-band	[36]
		1518	G-band	[36]
		2684	G’-band	[36]
	RhB-PEMS	1080	C-C stretching band	[50]
		1305	CH_2_ twisting	[50]
		1450	CH_2_ bending	[50]
		2800–3000	CH stretching vibrational modes	[50]

## Data Availability

Not applicable.
